# Effects of the Interleukin-6 Receptor Blocker Sarilumab on Metabolic Activity and Differentiation Capacity of Primary Human Osteoblasts

**DOI:** 10.3390/pharmaceutics14071390

**Published:** 2022-06-30

**Authors:** Annett Klinder, Janine Waletzko-Hellwig, Marie-Luise Sellin, Anika Seyfarth-Sehlke, Markus Wolfien, Franziska Prehn, Rainer Bader, Anika Jonitz-Heincke

**Affiliations:** 1 Research Laboratory for Biomechanics and Implant Technology, Department of Orthopedics, Rostock University Medical Centre, 18057 Rostock, Germany; annett.klinder@med.uni-rostock.de (A.K.); janine.waletzko-hellwig@med.uni-rostock.de (J.W.-H.); marie-luise.sellin@med.uni-rostock.de (M.-L.S.); anika.seyf@arcor.de (A.S.-S.); franziska.prehn@med.uni-rostock.de (F.P.); rainer.bader@med.uni-rostock.de (R.B.); 2 Department of Systems Biology and Bioinformatics, University of Rostock, 18057 Rostock, Germany; markus.wolfien@uni-rostock.de

**Keywords:** Interleukin 6, sarilumab, soluble IL-6 receptor, antagonism, human osteoblasts, osteogenic differentiation

## Abstract

Interleukin (IL-) 6 is a key factor in the inflammatory processes of rheumatoid arthritis. Several biologic agents target the IL-6 signaling pathway, including sarilumab, a monoclonal antibody that blocks the IL-6 receptor and inhibits IL-6-mediated cis- and trans-signaling. A careful analysis of the IL-6 signaling blockade should consider not only inflammatory processes but also the regenerative functions of IL-6. The purpose of this study was to investigate whether inhibition of the IL-6 receptors affects differentiation of human primary osteoblasts (hOB). The effects of sarilumab on viability and the differentiation capacity in unstimulated osteoblasts as well as after stimulation with various IL-6 and sIL6-R concentrations were determined. Sarilumab treatment alone did not affect the differentiation or induction of inflammatory processes in hOB. However, the significant induction of alkaline phosphatase activity which was observed after exogenous IL-6/sIL-6R costimulation at the highest concentrations was reduced back to baseline levels by the addition of sarilumab. The IL-6 receptor blockade also decreased gene expression of mediators required for osteogenesis and bone matrix maintenance. Our results demonstrate that concomitant administration of the IL-6 receptor blocker sarilumab can inhibit IL-6/sIL-6R-induced osteogenic differentiation.

## 1. Introduction

Interleukin (IL-) 6 influences bone-remodeling processes as it can enhance osteoclastic activity [1,2]. High levels of the cytokine trigger the expression of the receptor activator of nuclear factor-kappa Β ligand (RANKL) by osteoblasts and synovial cells which in turn activates the differentiation of osteoclasts [3]. Besides its osteoresorptive action, IL-6 promotes osteoblastic differentiation [4] and is also able to reduce osteoblastic proliferation [2]. The specific effects of IL-6 on the maturation of preosteoblasts into osteoblasts depend on the differentiation stage of osteoblasts. In recent years, the osteotropic function of IL-6 gained more importance [5], especially since it was proven that, after bone resorption, new bone formation is stimulated through an IL-6-dependent coupling mechanism [6]. Furthermore, a recent study showed that an increase in exercise capacity was mediated via IL-6 signaling in osteoblasts [7]. This highlights that IL-6 induces a wide variety of effects in the local bone environment.

After the binding of IL-6 to the membrane-bound IL-6 receptor (IL-6R), the IL-6/IL-6 receptor complex interacts with the glycoprotein (gp)130 subunit to induce IL-6-specific signaling [8]. Receptor–ligand interaction leads to phosphorylation of tyrosine residues on gp130 by Janus kinases (JAKs) and subsequently to the activation of various members of the signal transducer and activator of transcription (STAT) family as well as the mitogen-activated protein kinase (MAPK) signaling pathway [1,3]. The downstream transduction via IL-6R within the cell is mediated via cis-signaling. As a result, IL-6 acts as a regenerative and protective mediator. The membranous receptor is expressed mainly on T- and B-cells, monocytes, osteocytes, and osteoblasts [9,10]. Additionally, IL-6R exists as a soluble form. The soluble form of IL-6R can be generated by two different mechanisms: by cleavage of the extracellular domain of IL-6R, or by differential splicing [1]. This soluble IL-6 receptor is released into serum or synovial fluid by monocytes or endothelial cells and induces a variety of cells to respond to IL-6 stimuli via stimulating the gp130 unit [10]. The transduction via the soluble form of IL-6R is called trans-signaling and is mainly responsible for the proinflammatory activity of IL-6 [10]. Soluble gp130 (sgp130) represents the naturally occurring inhibitor of IL-6 trans-signaling, whereas IL-6 cis-signaling is unaffected by sgp130 [11,12]. The IL-6/soluble IL-6R receptor complex stimulates cells, which are also unresponsive to IL-6 alone and exhibit a lack of IL-6R [13], suggesting that trans-signaling may not only induce inflammation. In contrast to other studies that presumed that osteoblasts express membrane-bound IL-6R, McGregor et al. postulated that the osteoblastic IL-6R is nonfunctional. They assumed that the IL-6-associated signaling cascades in osteoblasts are induced only via the soluble receptor, which can be secreted by osteocytes [14].

IL-6 is also known as one of the most proinflammatory key factors in rheumatoid arthritis (RA). In RA, IL-6 is mainly synthesized by cells of the innate immune systems, e.g., monocytes and neutrophils. IL-6 synthesis is induced in these cells after Toll-like receptor (TLR) activation [10]. Additionally, osteoblasts, chondrocytes, and synovial fibroblasts were reported to express IL-6 [9]. Different biologic agents target the IL-6 pathway. These binds either directly the IL-6 cytokine (sirukumab, olokizumab, and clazakizumab) or the IL-6R (tocilizumab, sarilumab) [3,10,15]. Sarilumab is a monoclonal antibody that targets the membrane-bound and the soluble form of the IL-6 receptor and thus inhibits IL-6-mediated signal transduction in cells [16]. In patients with RA, sarilumab demonstrates a clear superiority over TNFα blocker in MTX intolerance and shows a similar safety profile compared to other IL-6 blockers [10]. The inhibition of the cis- and trans-signaling pathways by sarilumab reduces the inflammation and results in protection from bone and cartilage destruction in RA [9]. However, based on the aforementioned variety of IL-6-mediated effects, inhibition of IL-6 signaling might impact further processes in bone metabolism. Thus, a careful analysis of bone-remodeling events is required to determine the consequences of sarilumab administration. Research should focus on the effects of IL-6 signaling in the different bone cells. It must be analyzed whether IL-6R inhibition also influences the regenerative and protective function of IL-6 in human osteoblasts. Therefore, the purpose of this in vitro study was to evaluate whether the inhibition of the IL-6 receptor by sarilumab is associated with reduced osteoblastic differentiation and lower extracellular matrix accumulation.

## 2. Materials and Methods

### 2.1. Osteoblastic Cell Culture and Stimulation Experiments

Human primary osteoblasts were isolated from the femoral heads of patients undergoing primary total hip arthroplasty according to a well-established protocol [17]. The corresponding samples were collected after obtaining informed consent from the patients and approval from the local ethics committee (registration number: A 2018-0234, approval date: 10 December 2018) and were used for cell isolation under sterile conditions.

The isolated cells were cultured under standard cell culture conditions (5% CO_2_ and 37 °C) in calcium-free Dulbecco’s Modified Eagle Medium (DMEM, PAN-Biotech, Aidenbach, Germany) containing 10% fetal calf serum (FCS, PAN-Biotech), 1% amphotericin B, 1% penicillin–streptomycin, and 1% HEPES buffer (all: Sigma-Aldrich, Munich, Germany). The absence of calcium in the cell culture medium suppresses the mineralization of the osteoblastic cells and the development of bone-like nodules. This allows the expansion of the osteoblasts up to passage 3 and ensures the continued proliferation of these cells. Ascorbic acid (final concentration: 50 µg/mL), β-glycerophosphate (final concentration: 10 mM), and dexamethasone (final concentration: 100 nM) (all: Sigma-Aldrich, Munich, Germany) were added to the cell culture medium to maintain the osteogenic phenotype [17]. For the mineralization assays, the cell culture medium was additionally supplemented with calcium chloride dihydrate (CaCl_2_·2xH_2_O, final concentration: 1.8 mmol/L; Merck KGaA, Darmstadt, Germany).

Before performing the cell culture experiments, the osteogenic phenotype was checked by alkaline phosphatase staining (fuchsin + substrate-chromogen (DAKO, Hamburg, Germany) and determination of mRNA transcripts of Col1A1, ALPL, and BGLAP (Figure S1). A separate experimental set-up investigated the change in these markers from isolation up to passage 3 (Figure S2a–c). There was a significant increase in Col1A1 gene expression in the cultivated cells compared to the freshly isolated cells; however, there were no differences between passages 1, 2, and 3. A similar effect was observed for ALPL, but here, the changes were not significant. The gene expression of BGLAP was not significantly affected by cultivation. The data from these analyses showed that the primary osteoblasts in our cell culture, while still continuously proliferating, represented a rather mature osteoblastic phenotype after their cultivation up to the third passage in osteogenic medium (≥3 weeks). Moreover, the expression of genes involved in IL-6 signaling, e.g., IL6 and IL6ST, did not change over the cell culture passages, nor did the release of IL-6 protein into the cell culture supernatant (Figure S2d–f). The unaltered gene expression from passage 1 to passage 3 shows that osteoblasts in the third passage are as suitable for our experiments as cells cultivated for shorter periods.

For all cell culture experiments, osteoblasts were seeded in the third passage in 48-well cell culture plates with 20,000 cells per well and cultured for 24 h under standard cell culture conditions. This was followed by stimulation with IL-6 (Peprotech, Hamburg, Germany), rh sIL-6R (CD126; ImmunoTools, Friesoythe; Germany), and sarilumab (Kevzara^®^, Sanofi-Aventis, Montpellier, France). Before cells were stimulated with IL-6 and sIL-6R, basal concentrations of both mediators were determined by enzyme-linked immunosorbent assay (ELISA) according to the manufacturer’s instructions (IL-6: Invitrogen™ eBioscience™ Human IL-6 ELISA Ready-SET-Go!™ Kit, ThermoFisher Scientific, Waltham, MA, USA; sIL-6R: Human IL-6 Receptor ELISA Kit, Abcam, Berlin, Germany). Moreover, the release of soluble gp130 (sgp130) was analyzed using the Human sgp130 ELISA Kit (Sigma-Aldrich).

The respective concentrations for stimulation experiments were: (a) 0, 1, 10, and 50 ng/mL for IL-6 and (b) 5 and 50 ng/mL for recombinant human sIL-6R. Based on continuous dosing with 200 mg every two weeks as recommended for human application, a sarilumab concentration of 250 nM (40 µg/mL) was considered for the cell culture experiments. This dosing regime was associated with a significant improvement in symptoms in RA patients [18]. The concentration in the cell culture experiments was based on the maximum concentration (Cmax) in serum as measured in pharmacokinetic studies of sarilumab-treated patients [19,20]. However, in vitro inhibition of IL-6 signaling was already achieved by much lower concentrations of sarilumab. Xu et al. (2021) reported an IC50 of 0.226 nM in a proliferation assay in DS-1 cells and an IC50 of 0.146 nM in STAT3 signaling in HepG2/STAT3-Luc cells [21]. Therefore, lower concentrations (0.0625 nM, 0.625 nM, and 6.25 nM) of sarilumab were additionally used to validate the effect by comparison to the maximum dose. Exposure times varied between 24 h, 72 h, 168 h, and 336 h depending on the experimental setup.

### 2.2. Determination of Cell Morphology, Viability, and Osteoblastic Differentiation Capacity

Osteoblastic viability was determined after 24 h or 72 h by water-soluble tetrazolium salt (WST)-1 assay (Takara Bio, Saint-Germain-en-Laye, France). The respective stimulation medium was removed, and cells were washed with phosphate-buffered saline (PBS, Sigma-Aldrich, Munich, Germany). Subsequently, the cells were incubated with the WST-1 reagent in a ratio of 1:10 with cell culture medium. After 45 min at 37 °C and 5% CO_2_, 100 µL aliquots were transferred in duplicate to a 96-well plate to measure absorbance at 450 nm (reference: 630 nm) in a microplate reader (Tecan, Maennedorf, Switzerland). WST-1 reagent in medium without cells served as a blank.

Cell morphology was determined via actin stain and DAPI counterstain. For this purpose, osteoblasts (10,000 cells/well) were seeded into chamber slides, allowing adherence over a period of 24 h. Afterwards, cells were stimulated with 50 ng/mL IL-6 + 50 ng/mL sIL-6R ± 250 nM sarilumab. After 72 h, the medium was removed, and cells were washed with PBS and fixed with 4% paraformaldehyde at room temperature for 10 min. Fixed cells were washed with PBS, and the cell membrane was permeabilized with 0.5% Triton-X (Sigma-Aldrich, Munich, Germany) for 5 min at room temperature. Cells were washed again with PBS and incubated with 100 nM Acti-stain 488 Fluorescent Phalloidin (Cytoskeleton, Denver, CO, USA) for 30 min in the dark. Stained cells were washed with PBS and treated with diamidino-2-phenylindole dihydrochloride (DAPI) for 5 min at room temperature. For microscopic evaluation, the CytoViva^®^ Enhanced Darkfield microscope system (CytoViva, Inc., Auburn, AL, USA) and a 60× oil objective were used. Emission of the Phalloidin-stained actin filaments at 500 nm resulted in a green fluorescence for the cytoskeleton. Cell nuclei (blue fluorescence) were visualized at a wavelength of 461 nm. Both images were taken from the same spot and layered upon each other with the help of an image-processing software (Adobe Photoshop CS6, Adobe Systems Software Ireland Ltd., Dublin, Ireland). Additionally, cells at the same spot were visualized in darkfield illumination. Thus, intracellular vesicles or large endosomes or granules can be detected without further staining.

For quantification of osteoblastic differentiation, we determined the de novo biosynthesis of collagen type 1 (c-terminal propeptide of collagen type 1 (C1CP, MicroVue™ CICP EIA, QUIDEL, Quidel Corporation, San Diego, CA, USA)) and osteopontin (OPN, Human Osteopontin ELISA Kit, Abcam, Cambridge, UK) in the supernatants of stimulated cells via ELISA. The biochemical assays were performed according to the manufacturer’s instructions. Absorbance was measured at 405 nm using a microplate reader (Tecan). Protein values were normalized to total protein, which was quantified via Qubit Protein Assay Kit and Qubit 1.0 (both: Thermo Fisher Scientific, Waltham, MA, USA) according to the protocol of the manufacturer.

For quantification of enzymatic alkaline phosphatase (ALP) activity, cells were washed twice in TRIS buffer and lysed in aqua dest. containing 1% Triton-X (Sigma-Aldrich, Munich, Germany) and 1% phenylmethylsulfonyl fluoride (PMSF) for 10 min at room temperature. Afterward, the cell lysates were treated with 1 mM p-nitrophenyl phosphate, 100 mM 2-amino-2-methyl-1-propanol, and 5 mM MgCl_2_ in distilled water for one hour at 37 °C. After stopping the reaction with 2 M NaOH, absorption of 405 nm was measured on a microplate reader (Tecan).

### 2.3. Gene Expression Analysis

For gene expression analyses, total RNA was isolated by column purification (peqGOLD micro RNA Kit; VWR, Darmstadt, Germany) followed by elution in RNase-free water. At least 50 ng of RNA of each donor was transcribed into cDNA using the High-Capacity cDNA Reverse Transcription Kit (Applied Biosystems, Forster City, CA, USA) according to the manufacturer’s instructions. The resulting cDNA was diluted 1:1 with RNase-free water and stored at −20 °C. For semiquantitative real-time PCR (qRT-PCR) 1 µL cDNA was mixed with 9 µL of master mix containing the primer pairs listed in Table 1 and the SybrGreen InnuMix (Analytik Jena, Jena, Germany). Each sample was tested in duplicate. QRT-PCR was performed according to the following protocol: 2 min at 95 °C, followed by 40 times of rotation of denaturation of 5 s at 95 °C, and annealing/elongation for 25 s at 60–65 °C. A cycle threshold (Ct) of 30 was set as a limit. The relative expression of target mRNA of stimulated cells was calculated using 2^(−ΔΔCt)^ method (% unstimulated control) [22].

### 2.4. Transcriptome Analysis

Transcriptome analysis was performed by ATLAS Biolabs (Berlin, Germany) using extracted and pooled total RNA from seven independent donors. Due to the low amount of purified target cells per individual patient, pooling of patient material was necessary to compensate for insufficient sample RNA (*n* = 1). For this purpose, the lysed cells from all seven donors (per stimulation experiment) were loaded onto a single column of the above-mentioned extraction kit to obtain the pooled RNA. Differences in gene expression levels of at least 10,135 genes were determined based on the gene level fold-change (<−2 or >2) on a Clariom D Human Array (Affymetrix, Applied Biosystems, Forster City, CA, USA). Analysis of microarray data was performed with the Transcriptome Analysis Console Software from Thermo Fisher (Version 4.0.1, Waltham, MA, USA). To confirm the results obtained from the transcriptome analysis, a follow-up analysis of gene expression levels of selected mRNA transcripts was conducted using three independent osteoblastic donors (see Section 2.3).

### 2.5. Mineralization Assay

Quantification of in vitro mineralization was realized with the OsteoImage™ Mineralization Assay (Lonza, Walkersville, MD, USA). For this purpose, human osteoblasts (5000 cells/well) were seeded into Thermo Scientific™ Nunc Black-walled 96-well plates (ThermoFisher Scientific, Waltham, MA, USA) using the same medium as indicated in Section 2.1 but supplemented with calcium chloride dihydrate (CaCl_2_·2xH_2_O, final concentration: 1.8 mmol/L; Merck KGaA, Darmstadt, Germany). After 24 h, the medium was replaced, and cells were stimulated with 50 ng/mL IL-6 + 50 ng/mL sIL-6R ± 250 nM sarilumab in the calcium-supplemented medium over a period of 7 and 14 days. Unstimulated cells served as controls. Afterward, cells were fixed with 4% paraformaldehyde, and staining of the hydroxyapatite portion of bone-like nodules deposited by the osteoblasts was performed according to the manufacturer’s protocol. Fluorescence signals of mineralization were detected at excitation/emission wavelengths of 492/520 nm using the microplate reader (Tecan). Afterward, the deposition of bone-like nodules was visualized via fluorescence microscopy (Nikon ECLIPSE TS100, Nikon GmbH, Duesseldorf, Germany) using a 40× objective.

### 2.6. Graphical Illustration and Statistics

For the experiments, osteoblasts from a total of 39 individual human donors (male, *n* = 15: 69.6 years ± 9.7 years; female, *n* = 24: 70.8 ± 13.5 years) were used. However, due to the low availability of donor-specific osteoblasts, it was not feasible to include each donor in each experiment. Therefore, osteoblasts from a minimum of three independent donors (in duplicate) were used for the experiments. Where feasible, data were also analyzed separately for female and male donors (see Supplementary Figures). If not otherwise stated, data are depicted as box plots with medians, interquartile ranges, and minimum/maximum values using GraphPad Prism 7.02 (Graphpad Software, San Diego, CA, USA). Statistical analyses were performed with GraphPad Prism 7.02. The normality of the data was proved via the Shapiro–Wilk test. Different stimulation groups were compared either with Friedman’s test with Dunn’s multiple comparisons test as a post hoc test or with repeated-measures two-way ANOVA or ordinary two-way ANOVA with Bonferroni’s multiple comparison post hoc test, as required. A *p*-value of less than 0.05 was defined as statistically significant.

## 3. Results

### 3.1. Influence of Exogenous IL-6 on the Secretion of sIL-6R and sgp130

In a preliminary analysis, the intrinsic release of IL-6 by human osteoblasts was determined via ELISA and showed low IL-6 protein concentrations (mean ± standard deviation: 71.5 ± 10.1 pg/mg). Therefore, experiments with increasing IL-6 concentrations (0 ng/mL, 1 ng/mL, 10 ng/mL, and 50 ng/mL) were carried out to identify distinct effects of IL-6 alone. The analyses of the release of soluble IL-6R (sIL-6R) and soluble gp130 (sgp130) in response to the different IL-6 concentrations showed that human osteoblasts secreted low amounts of sIL-6R compared to sgp130. There were no significant differences in the release by IL-6 stimulated and unstimulated osteoblasts. The release of sgp130 was generally approximately 50 times higher than the release of sIL-6R in the osteoblasts. Here, unstimulated osteoblasts showed the highest sgp130 concentrations compared to cells stimulated with exogenous IL-6. However, increasing IL-6 showed no additional effects (Table 2).

### 3.2. Influence of Sarilumab on Osteoblastic Viability and Gene Expression

**Figure 1:**
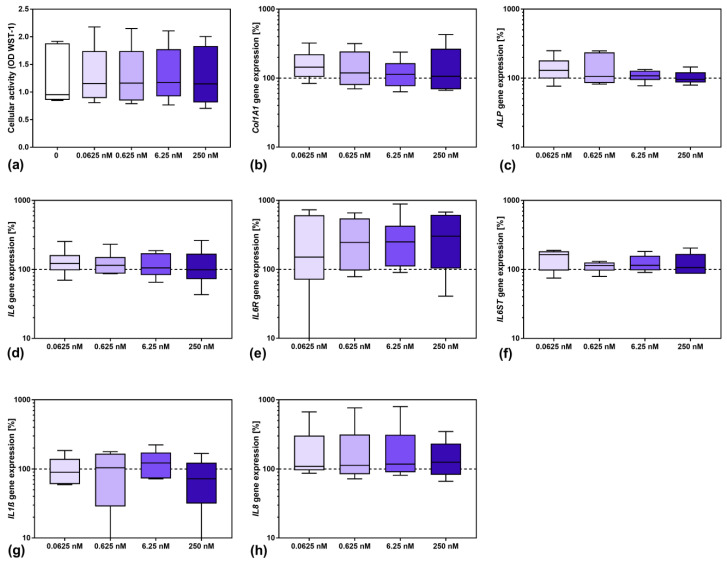
Influence of sarilumab on osteoblastic viability (**a**) as well as gene expression of osteogenic differentiation markers (**b**,**c**), and mediators involved in inflammation (**d**–**h**). (**a**) Cell viability was tested via water-soluble tetrazolium salt (WST-)1 assay. (**b**–**h**) Total RNA of human osteoblasts was isolated and gene expression patterns of the different markers were determined. Results were calculated by 2^−ΔΔCt^ method (% unstimulated control). All data are shown as box plots (*n* = 6 independent female donors (passage 3); mean age: 77.5 ± 11.4 year; apart from gp130 with *n* = 5 individual female donors (passage 3); mean age: 77.8 ± 12.8 year). Statistical significance was determined via the Friedman’s test with Dunn’s multiple comparisons test as post hoc test.

Before experiments on the effect of sarilumab in IL-6-stimulated osteoblasts could be performed, it was necessary to investigate its influence on cells. Therefore, cells were treated with different concentrations of sarilumab to analyze its effect on osteoblastic viability (Figure 1a) and gene expression (Figure 1b–h). For all investigated parameters, no significant differences were observed between untreated and sarilumab-treated cells. The induction of osteogenic differentiation markers was unaffected by the increasing sarilumab concentrations (Figure 1b,c). Furthermore, supplementation with sarilumab alone did not affect IL6 (Figure 1d) and IL6ST (Figure 1f) gene expression, whereas a slight increase in IL6R transcripts (Figure 1e) was detected. Furthermore, there was no significant effect of sarilumab on the induction of the proinflammatory mediators IL1β and IL8 (Figure 1g,h).

### 3.3. Influence of Stimulation with Exogenous IL-6 and sIL-6R on Osteoblastic Viability and Differentiation

**Figure 2:**
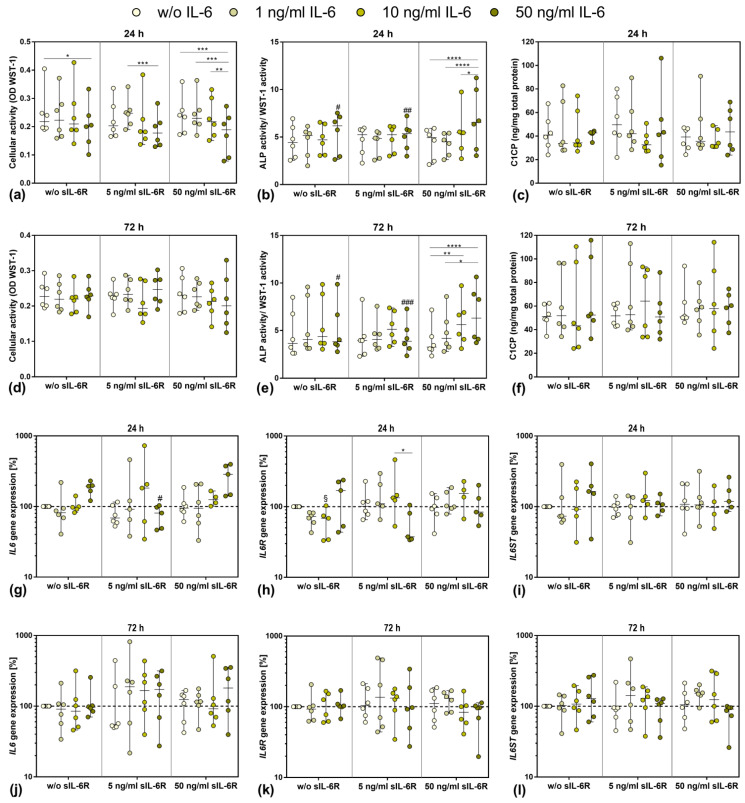
Influence of different IL-6 and sIL-6R concentrations on osteoblastic viability and differentiation capacity after 24 h (**a**–**c**,**g**–**i**) and 72 h (**d**–**f**,**j**–**l**) of exposure. (**a**,**d**) Cell viability was tested via water-soluble tetrazolium salt (WST) 1 assay (*n* = 6 independent donors (passage 3; female: *n* = 1, male: *n* = 5); mean age: 71.5 ± 11.6 year). (**b**,**e**) Alkaline phosphatase (ALP) activity of osteoblasts, which was quantified via hydrolysis of p-nitrophenylphosphate (*n* = 6 independent donors (passage 3; female: *n* = 1, male: *n* = 5); mean age: 71.5 ± 11.6 year). (**c**,**f**) Release of c-terminal propeptide of collagen type 1 (C1CP) in the cell culture supernatant (*n* = 6 independent donors (passage 3; female: *n* = 1, male: *n* = 5); mean age: 71.5 ± 11.6 year). (**g**–**l**) Gene expression analysis of IL6, IL6R, and IL6ST in human osteoblasts (24 h: *n* = 4–6 independent donors, 72 h: *n* = 6 independent donors (passage 3; female: *n* = 1, male: *n* = 5); mean age: 71.5 ± 11.6 year). Results were calculated by 2^−ΔΔCt^ method (% unstimulated control). All data are shown as box plots. Statistical significance was determined via two-way ANOVA or repeated-measures two-way ANOVA with Bonferroni’s post hoc test: * *p* < 0.05, ** *p* < 0.01; *** *p* < 0.001, **** *p* < 0.0001 (significance between IL-6 concentrations); ^§^ *p* < 0.05 (significantly different from sample with same IL-6 concentration at 5 ng/mL sIL-6R); ^#^ *p* < 0.05; ^##^ *p* < 0.01; ^###^ *p* < 0.001 (significantly different from sample with same IL-6 concentration at 50 ng/mL sIL-6R).

Since the baseline secretion of sIL-6R by human osteoblasts was already low and did not change following stimulation with different IL-6 concentrations, the supplementation with sIL-6R seemed to be necessary to analyze the effects of trans-signaling of IL-6. Thus, IL-6 concentration-dependent experiments were carried out in the presence or absence of 5 ng/mL or 50 ng/mL sIL-6R over a period of 24 h or 72 h. Overall, the presence of sIL-6R in IL-6-unstimulated cells did not affect either cell viability or osteoblastic differentiation. For osteoblasts treated with IL-6 alone, only a significant reduction in cell viability was visible for the highest IL-6 concentration after 24 h (*p* = 0.0441 vs. 0 ng/mL IL-6; Figure 2a). The same effect was observed in the presence of 5 ng/mL sIL-6R and 50 ng/mL IL-6 (*p* = 0.0008 vs. 1 ng/mL IL-6). When cells were costimulated with 50 ng/mL sIL-6R, the reduction in metabolism was significant for the highest IL-6 concentration compared to the other IL-6 concentrations (*p* = 0.0005 vs. 0 ng/mL IL-6; *p* = 0.0003 vs. 1 ng/mL IL-6; *p* = 0.0027 vs. 10 ng/mL IL-6, respectively). Although a similar trend was visible after 72 h, no significant differences in cell viability could be determined (Figure 2d).

ALP activity of human osteoblasts normalized to WST-1 activity was only affected at supplementation with the highest concentration of sIL-6R (50 ng/mL). At this concentration, ALP activity/WST-1 activity increased in a dose-dependent manner with the increase in exogenous IL-6 concentration (Figure 2b,e). This significant increase was observed after 24 h (w/o IL-6 vs. 50 ng/mL IL-6 *p* < 0.0001, 1 ng/mL IL6 vs. 50 ng/mL IL-6 *p* < 0.0001 and 10 ng/mL IL-6 vs. 50 ng/mL IL-6 *p* = 0.0410, respectively) and 72 h (w/o IL-6 vs. 10 ng/mL IL-6 *p* = 0.0059, w/o IL6 vs. 50 ng/mL IL-6 *p* < 0.0001 and 1 ng/mL IL-6 vs. 50 ng/mL IL-6 *p* = 0.0105, respectively). Collagen 1 protein (C1CP) biosynthesis did not appear to be significantly affected in the stimulation groups. High donor-dependent differences were evident here. Over time, C1CP accumulation occurred in some experimental groups, but again, no differences were noticeable (Figure 2c,f).

### 3.4. Gene Expression of IL6, IL6R, and IL6ST after Exposure to Exogenous IL-6 and sIL-6R

To further analyze the impact of exogenous IL-6 and sIL-6R costimulation, gene expression rates of relevant mediators involved in IL-6 signaling were determined (Figure 1g–l). Compared to unstimulated cells, IL6 gene expression was induced after 24 h following stimulation with 50 ng/mL IL-6 either in the absence or presence of 50 ng/mL sIL-6R. Contrarily, IL6 mRNA was significantly reduced after treatment with the highest IL-6 concentration in the presence of 5 ng/mL sIL-6R (*p* = 0.0156) (Figure 2g). After 72 h, IL6 gene expression levels were unaffected in osteoblasts treated with IL-6 alone. Costimulation with 5 ng/mL sIL-6R caused a trend towards increased IL6 gene expression in the cells, but without significant changes between the applied IL-6 concentrations. Only costimulation with the highest IL-6 and sIL-6R concentrations led to enhanced IL6 mRNA transcripts. However, due to high interindividual differences, the values did not reach statistical significance (Figure 2j).

In the absence of sIL-6R, a downregulation of IL6R mRNA was observed for osteoblasts after 24 h when stimulated with lower IL-6 concentrations (10 ng/mL IL-6: *p* = 0.0218 w/o sIL-6R vs. 5 ng/mL sIL-6R). For the highest IL-6 concentration, a simultaneous increase in IL6R expression levels could be demonstrated compared to IL6 mRNA. The same effect was observed for 5 ng/mL sIL-6R costimulation. IL-6 costimulation with 50 ng/mL sIL-6R resulted in a slight concentration-dependent increase in IL6R transcripts; however, for the highest IL-6 concentration, IL6R gene expression was unaffected (Figure 2h). After 72 h, neither the sole IL-6 stimulation nor the costimulation with 5 ng/mL sIL-6R affected IL6R gene expression. Only the presence of 50 ng/mL sIL-6R led to an IL-6 concentration-dependent downregulation of IL6R transcripts (Figure 2k).

Gene expression of IL6ST was predominantly unaffected by the respective stimulations at 24 h. However, an upregulation of IL6ST mRNA could be observed following stimulation with the highest IL-6 concentration in the presence or absence of 50 ng/mL sIL-6R (Figure 2i). After 72 h, IL6ST gene expression was almost unaffected when stimulated with IL-6 alone, as well as after costimulation of IL-6 and 5 ng/mL sIL-6R. However, the highest sIL-6R concentration resulted in an IL-6 concentration-dependent decrease in IL6ST mRNA (Figure 2l).

### 3.5. Viability and Differentiation Capacity of Human Osteoblasts after Exposure to IL-6, sIL-6R, and Sarilumab Treatment

Human osteoblasts were exposed to exogenous IL-6 (10 ng/mL and 50 ng/mL), sIL-6R (50 ng/mL), and sarilumab (250 nM) over a period of 72 h to investigate the influence on osteoblastic morphology, viability, and differentiation capacity.

**Figure 3:**
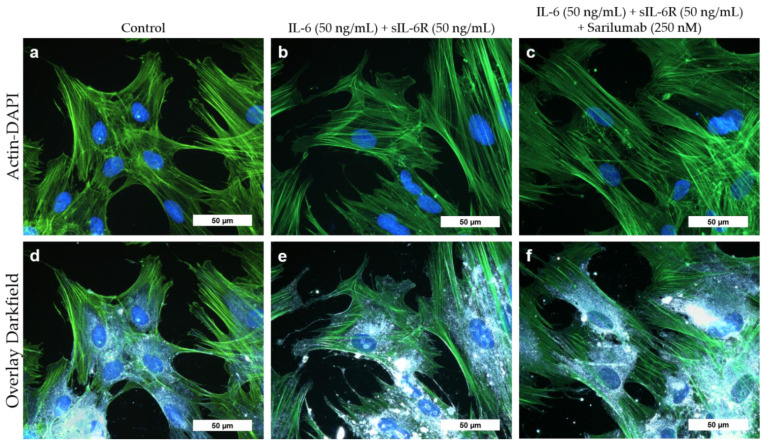
Cell morphology of human osteoblasts (passage 3, male donor, 68 year) after exposure to IL-6, sIL-6R ± sarilumab for 72 h. (**a**–**c**) Fluorescence stain of the actin cytoskeleton (green fluorescence) and cell nuclei (blue fluorescence). (**d**–**f**) Overlay of the stained actin cytoskeleton, nuclei, and enhanced darkfield microscopy to visualize intra- and extracellular structures. Bar: 50 µm.

Cell morphology of osteoblasts did not change when cells were exposed to IL-6 (50 ng/mL) and sIL-6R (50 ng/mL) with or without sarilumab (250 nM). The cytoskeleton was uniformly expressed throughout the cell in all experimental groups (Figure 3a–c). Darkfield illumination was used to visualize granular structures within the cells (Figure 3d–f).

**Figure 4:**
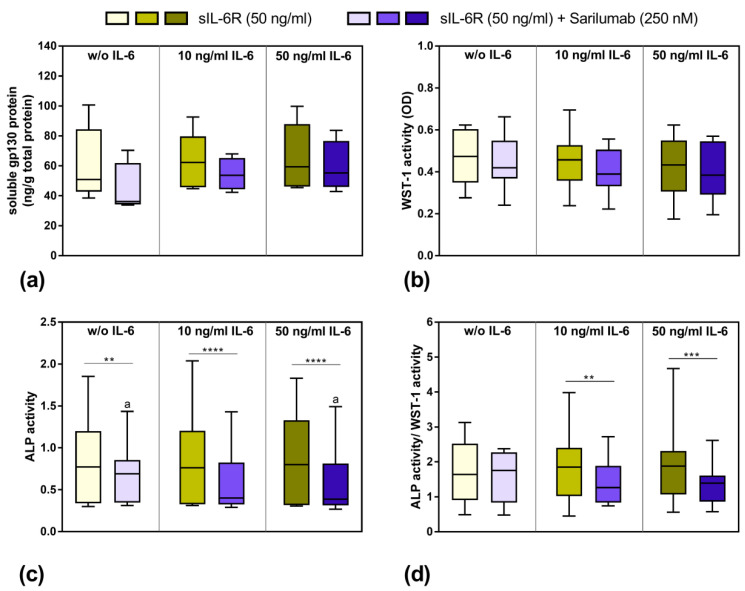
Release of soluble gp130 (sgp130), osteoblastic viability, and alkaline phosphatase (ALP) activity after exposure to IL-6, sIL-6R ± sarilumab for 72 h. (**a**) Secreted sgp130 was determined in supernatants of exposed osteoblasts in passage 3 (*n* = 4 independent donor (female: *n* = 3, male: *n* = 1; mean age: 77.5 ± 5.1 year)—*n* = 5 independent donors (female: *n* = 4, male: *n* = 1); mean age: 77.8 ± 4.4 year). (**b**) Cell viability was tested via water-soluble tetrazolium salt (WST-)1 assay (*n* = 12 independent donors in passage 3 (female: *n* = 8, male: *n* = 4); mean age: 67.3 ± 16.1 year). (**c**) Alkaline phosphatase (ALP) activity of osteoblasts, which was quantified via hydrolysis of p-nitrophenyl phosphate (*n* = 12 independent donors in passage 3 (female: *n* = 8, male: *n* = 4); mean age: 67.3 ± 16.1 year). (**d**) ALP activity related to metabolic activity (*n* = 12 independent donors in passage 3 (female: *n* = 8, male: *n* = 4); mean age: 67.3 ± 16.1 year). All data are shown as box plots. Statistical significance was determined via repeated-measures two-way ANOVA with Bonferroni’s post hoc test: ** *p* < 0.05; *** *p* < 0.001, **** *p* < 0.0001 (significantly different from samples with sarilumab costimulation); ^a^ *p* < 0.05 (significance between IL-6 concentrations).

Although the release of sgp130 by human osteoblasts showed a donor-dependent range of variability, the stimulation with 10 and 50 ng/mL IL-6 in the presence of sIL-6R seemed to slightly increase the release of this soluble glycoprotein. Interestingly, in the absence of exogenous IL-6 supplementation, the costimulation with sIL-6R and sarilumab decreased the protein concentration in osteoblasts compared to the treatment of the cells with sIL-6R in the absence of sarilumab. However, this effect was not statistically significant (Figure 4a). The viability of human osteoblasts was mainly unaffected by any of the combinations of IL-6, sIL-6R, and sarilumab treatments (Figure 4b). ALP activity was significantly reduced after the addition of sarilumab to the cell cultures (*p* = 0.0024, *p* < 0.0001 and *p* < 0.0001 for stimulation w/o IL-6, 10 ng/mL IL-6, and 50 ng/mL IL-6, respectively, in combination with sIL-6R and sarilumab compared to sIL-6R supplementation alone). While this effect occurred independently from the supplementation with exogenous IL-6, it was more pronounced when IL-6 was added. Thus, ALP activity was significantly lower in cells stimulated with sIL-6R, sarilumab, and 50 ng/mL IL-6 (*p* = 0.0236) compared to cells treated only with sIL-6R and sarilumab (Figure 4c). This was confirmed when ALP activity was normalized by dividing it by the values of metabolic activity (ALP activity/WST-1 activity). Here, the decrease in the values after the addition of sarilumab was only significant when the cells were simultaneously stimulated with exogenous IL-6 (*p* = 0.0027 and *p* = 0.0002 for 10 ng/mL IL-6 and 50 ng/mL IL-6, respectively, Figure 4d).

In contrast, treatment with IL-6, sIL-6R ± sarilumab did not affect C1CP synthesis (Table 3). Osteopontin release from cells was unaffected in the presence of sIL-6R, independent of whether the cells were treated with or without exogenous IL-6. While there was already no osteopontin release in one of five donors at costimulation of 50 ng/mL sIL-6R and 50 ng/mL IL-6, the addition of sarilumab suppressed the release of osteopontin by human osteoblasts in three (0 ng/mL IL-6), one (10 ng/mL), or two (50 ng/mL IL-6) of the four analyzed donors (Table 3). There was no concentration-dependent effect, and the changes were not significant.

### 3.6. Transcriptome Analysis of Sarilumab-Responsive Osteoblasts and Confirmation of Results by Independent qPCR Experiments

**Figure 5:**
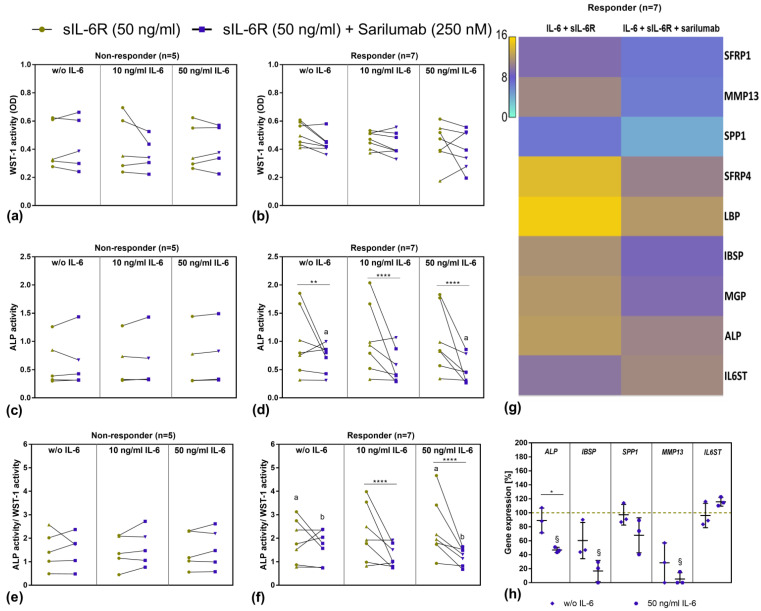
Overview of sarilumab-responsive and nonresponsive osteoblasts after 72 h stimulation with IL-6, sIL-6R ± sarilumab. (**a**,**b**) Cell viability was tested via water-soluble tetrazolium salt (WST-)1 assay. (**c**,**d**) ALP activity of osteoblasts, which was quantified via hydrolysis of p-nitrophenyl phosphate (pNpp). (**e**,**f**) ALP activity normalized to metabolic activity. All data are shown as single data points with connecting lines for the same cell donor (*n* = 7 independent donors (passage 3) for responder (female: *n* = 4, male: *n* = 3; mean age: 70.3 ± 6.0 year) and *n* = 5 independent donors (passage 3) for nonresponder (female: *n* = 4, male: *n* = 1; mean age 65.0 ± 24.0 year) with female donors (green dot and purple square) and male donors (green up-facing triangle and purple down-facing triangle) assigned separate symbols. Statistical significance was determined via repeated-measures two-way ANOVA with Bonferroni’s post hoc test: ** *p* < 0.01; **** *p* < 0.0001 (significantly different from samples with sarilumab costimulation); ^a,b^ *p* < 0.05 (same letters indicate significance between different IL-6 concentrations) (**g**) Overview of strongest regulated genes determined via transcriptome analysis. Here, RNA from a total of seven independent donors was pooled and extracted for gene expression analysis via Clariom D Human array. (**h**) Validation of selected genes that were regulated in transcriptome analysis. To this purpose, human osteoblasts of three independent donors in passage 3 (female: *n* = 1, male: *n* = 2); mean age: 76.7 ± 9.2 year) were treated with sIL-6R, sarilumab ± IL-6 over a period of 72 h. Gene expression patterns were calculated by 2^−ΔΔCt^ method (% unstimulated control). Data are shown as means ± SD. Statistical significance was determined via repeated-measures two-way ANOVA with Bonferroni’s post hoc test: * *p* < 0.05 (significance between IL-6 concentrations); ^§^ *p* < 0.05 (significantly different from samples without sarilumab co-stimulation). Abbreviations: SFRP: Secreted frizzled-related protein; MMP: Matrix metalloprotease; SPP1: Osteopontin; LBP: Lipopolysaccharide binding protein; IBSP: Integrin-Binding Sialoprotein; MGP: Matrix Gla protein; ALP: Alkaline Phosphatase; gp130: Glycoprotein 130.

Due to the relatively high variation in our data, we decided to analyze the data in detail by determining the effects in the single donors separately. The ALP activity/WST-1 activity after stimulation with 50 ng/mL sIL-6R and 50 ng/mL IL-6 as well as the change after addition of sarilumab did not depend on the age of the patients (Figure S3). There were also no differences in the stimulation response with regard to the gender of the donors (Figure S4). However, we observed that 7 out of 12 independent osteoblastic donors showed a strong response to stimulation with IL-6 and sIL-6R and subsequent inhibition by sarilumab, while the other five donors showed no differences either in cell viability (Figure 5a,b) or ALP activity (Figure 5c–f). Responders were identified as donor cells that showed considerably decreased ALP activity after treatment with sarilumab. For sarilumab-responsive cells, a distinct concentration-dependent effect of IL-6 stimulation was also present in ALP activity/WST-1 activity, which in turn led to a significant reduction (*p* = 0.0398) when cotreated with sarilumab (Figure 5e,f).

However, apart from the above-mentioned changes, we were not able to determine significant differences in gene expression patterns for IL6, IL6R, and Col1A1, as well as for BGLAP, OPG, and RANKL. To obtain a comprehensive overview and to identify transcriptional changes associated with the effect on ALP activity, a transcriptome analysis (Clariom D Human array) of the osteoblasts from the seven sarilumab-responsive donors (responders) was carried out. The results from the transcriptome analysis confirmed the previous findings that the addition of sarilumab did not change gene expression rates of IL6, IL6R, and collagen or other relevant osteogenic differentiation markers such as Runx2 or SP7 in IL-6/IL-6R-stimulated osteoblasts. However, it was shown that sarilumab treatment upregulated IL6ST (2.32-fold). With the exception of IL6ST, the altered gene expression manifested mainly in the downregulation of specific genes. Figure 5g summarizes the genes that were downregulated the strongest following sarilumab supplementation. The identified genes can be divided into four different groups: (a) genes responsible for the preservation of the bone matrix: Matrix Gla protein (MGP, involved in calcium deposition; 6.52-fold), integrin-binding sialoprotein (IBSP; 6.64-fold), osteopontin (SPP1; 5.84-fold); (b) genes responsible for bone degradation: Matrix metalloprotease (MMP)13 (22.69-fold); (c) genes involved in Wnt signaling: secreted frizzled-related protein (SFRP)1, and 4 (both: Wnt-receptor-binding antagonists; 5.84-fold and 13.2-fold); (d) genes involved in Toll-like receptor signaling: Lipopolysaccharide binding protein (LBP, pathogen detection; 10.37-fold).

Follow-up analysis with semiquantitative PCR using three individual donors was performed for selected transcripts to validate the results of the transcriptome analysis. The qPCR results from cells of three different donors, which were independent of those donors that were used in the transcriptome analysis, confirmed the results of the transcriptome analysis. It was shown that costimulation of IL-6 with sIL-6R and sarilumab led to significantly reduced transcripts of ALP (*p* = 0.0227), IBSP (*p* = 0.0169), and MMP13 (*p* = 0.0321) in these three different donors. In addition, a nonsignificant downregulation was detected for SPP1. As expected, mRNA transcripts of the IL6ST subunit were upregulated. Without exogenous IL-6 stimulation, a similar but nonsignificant trend was determined (Figure 5h).

### 3.7. Mineralization Capacity of Human Osteoblasts Treated with IL-6, sIL-6R ± Sarilumab

**Figure 6:**
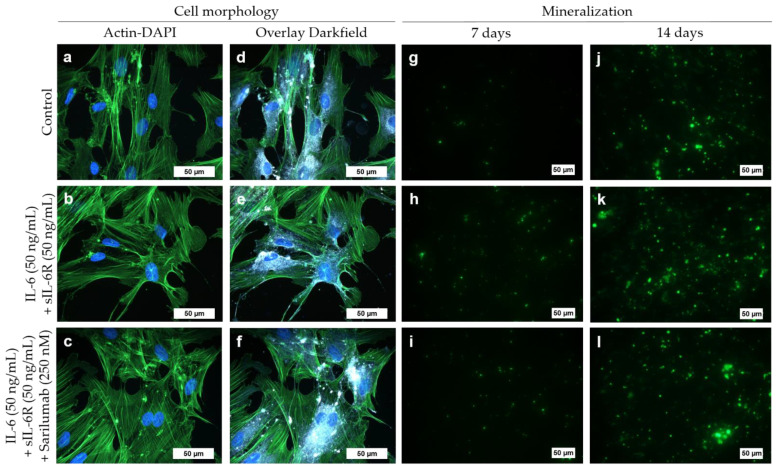
Cell morphology (**a**–**f**) and mineralization capacity (**g**–**l**) of human osteoblasts following exposure to IL-6, sIL-6R ± sarilumab over 72 h in calcium-supplemented medium (passage 3, male donor, 68 year). (**a**–**c**) Fluorescence stain of the actin cytoskeleton (green fluorescence) and cell nuclei (blue fluorescence; bar: 50 µm). (**d**–**f**) Overlay of the stained actin cytoskeleton, nuclei, and enhanced darkfield microscopy to visualize intra- and extracellular structures (bar: 50 µm). (**g**–**l**) Hydroxyapatite portion of bone-like nodules (green fluorescence) deposited by the osteoblasts after 7 and 14 days (bar: 50 µm).

To determine the mineralization capacity of human osteoblasts during treatment with IL-6, sIL-6R, and sarilumab, cells were cultured in calcium-supplemented medium for 7 and 14 days. There were no morphological changes in osteoblasts cultivated in calcium-containing medium (Figure 6a–c) compared to osteoblasts grown in calcium-free medium (Figure 4). The osteoblasts exhibited a uniformly expressed cytoskeleton. In addition, dark-field microscopy revealed multiple bright-illuminated extracellular deposits (Figure 6d–f).

**Figure 7:**
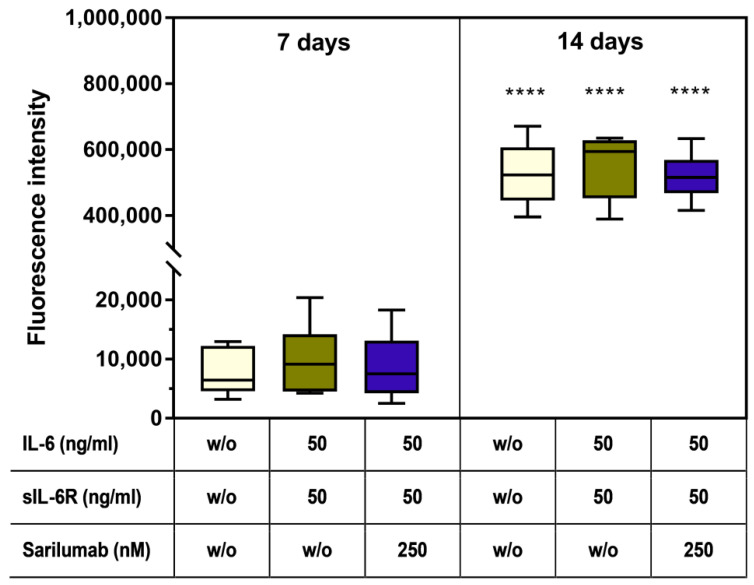
Mineralization capacity of human osteoblasts (*n* = 6 independent donors in passage 3 (female: *n* = 3, male: *n* = 3; mean age: 65.3 ± 7.8 year). Cells were stimulated with IL-6, sIL-6R ± sarilumab over a period of 7 or 14 days in calcium-supplemented medium. Afterward, deposition of the hydroxyapatite portion of bone-like nodules was quantified via OsteoImage™. Fluorescence signals of mineralization were detected at excitation/emission wavelengths of 492/520 nm using a microplate reader. Statistical significance was determined via repeated-measures two-way ANOVA with Bonferroni’s post hoc test: **** *p* < 0.0001 (significantly different from samples of the same stimulation group at 7 days).

Already after seven days, a deposition of bone-like nodules could be detected in all experimental groups (Figure 6g–i). Furthermore, after 14 days, a significant increase (all stimulation groups: *p* < 0.0001) in hydroxyapatite deposition was detected (Figure 6j–l and Figure 7). There were no significant differences between the treatment groups or compared to the untreated control (Figure 6h,k and Figure 7). Female donors showed an overall reduced mineralization at day 7 compared to male donors (two-way ANOVA: *p* = 0.0048 for factor “gender”); however, pairwise comparison of male and female donors in the different treatment groups by Bonferroni’s post hoc test revealed no significant differences. The gender-specific effect at day 7 was independent of the stimulation of the cells (two-way ANOVA: *p* = 0.6481 for factor “treatment” and *p* = 0.9342 for interaction of both factors) and completely disappeared at day 14 (two-way ANOVA: *p* = 0.7187, *p* = 0.8334, and *p* = 0.9636 for factors “gender”, “treatment”, and interaction, respectively; Figure S5).

## 4. Discussion

The main purpose of our study was to evaluate the effect of IL-6 and its antagonism on the differentiation capacity of human osteoblasts. Thus, the key findings were as follows. (i) Human osteoblasts synthesize IL-6, sgp130, and, only to a very low extent, sIL-6R. (ii) High concentrations of exogenous IL-6 induce osteogenic differentiation only in combination with exogenous sIL-6R. (iii) The addition of sarilumab alone did not affect the gene expression of osteogenic differentiation markers and proinflammatory mediators. Under costimulation with IL-6 and sIL-6R, sarilumab can decrease IL-6/sIL-6R-induced osteogenic differentiation capacity. (iv) As an early cell response, sarilumab has a potential downstream regulatory effect on osteogenic differentiation pathways.

The results of our study demonstrate that human osteoblasts tolerate high IL-6 concentrations. The intrinsically released concentration of IL-6 in the osteoblasts was on average 71 pg/mg. However, even a 200-fold higher concentration of exogenous IL-6 had no considerable effect on cell viability and osteogenic differentiation. This confirms the observation by Vermes et al. (2012), who hypothesized that human osteoblasts are resistant to autocrine-produced IL-6 [1]. It was further described that human osteoblasts can respond rapidly to IL-6 by secreting the nonfunctional cell surface IL-6R [1]. Similarly, IL6R was overexpressed after 24 h in our study, especially at the highest IL-6 concentration. This was also associated with higher IL6ST transcript levels after 24 h and 72 h. However, the induction of IL6ST mRNA seems to have no significant impact on the induction of signaling pathways associated with osteogenic differentiation. Moreover, the release of sgp130 appears to be inversely related to the presence of IL-6 protein, as higher exogenous IL-6 concentrations led to a reduction in sgp130. It is known that gp130-mediated signaling in osteoblasts promotes bone formation [23]. Our data suggest that, in accordance with McGregor et al. (2019), activation of human osteoblasts occurs only via the solubilized IL-6 receptor [14]. A direct action of IL-6 on osteoblast bone formation, especially after the addition of sIL-6R, cannot be ruled out [24]. However, newer research suggests that IL-6 indirectly regulates bone formation by suppressing the expression of sclerostin—a bone formation inhibitor—in osteocytes via STAT3 signaling [25]. The involvement of osteocytes is supported by the fact that differentiated osteocytes are a source of sIL-6R while sIL-6R is not secreted by mature osteoblasts [14].

This is consistent with the very low release of sIL-6R by the primary osteoblasts in our study. The addition of sIL-6R seemed to be necessary to obtain a biological response to exogenously applied IL-6 [26]. Furthermore, it was shown that the addition of 5 ng/mL sIL-6R was not sufficient to induce a pronounced cell response and effects. Especially on ALP activity, an effect could only be achieved with higher sIL-6R concentrations. In accordance with research by Nishimura et al. (1998), we also observed that the IL-6/sIL-6R-mediated osteoblastic response was accompanied by an increase in ALP activity [27]. In bone metabolism, ALP influences mineralization and bone maintenance [28]. ALP catalyzes, for example, the dephosphorylation of OPN, which is an important regulator of biomineralization [28,29,30]. Phosphorylated OPN inhibits biomineralization by binding calcium ions via its phosphate residues. When OPN is dephosphorylated by ALP, it is no longer able to suppress hydroxyapatite deposition [31]. Despite the increasing ALP activity caused by IL-6/sIL-6R stimulation, no relevant changes were detected for osteopontin after stimulation. This indicates that synthesis of osteopontin was unaffected by IL-6. Still, as OPN protein quantification via the ELISA does not differentiate between phosphorylated and dephosphorylated OPN, an effect of the increased ALP activity cannot be ruled out. Interestingly, it was described that osteopontin may also be involved in inflammatory processes; however, the influence of ALP-induced dephosphorylation of osteopontin on associated inflammatory effects needs to be clarified [30].

Sarilumab is a monoclonal antibody that targets the membrane-bound and the soluble form of the IL-6 receptor, resulting in inhibition of IL-6-mediated signal transduction in cells [16]. In our study, it can be concluded that the inhibition of IL-6 receptors by sarilumab influences the intrinsically released IL-6 and its signal transduction in human osteoblasts. However, significant effects of sarilumab seem to be achieved only at the highest extracellular IL-6 concentrations. While there was no impact of age or gender, donor specificity of sarilumab action was observed. This is in accordance with clinical studies analyzing the efficacy of sarilumab in rheumatoid arthritis (RA). Here, it was reported that approximately 30% of patients did not show improvement in the American College of Rheumatology (ACR)/European League Against Rheumatism Classification Criteria for RA and can be considered as nonresponders [18]. The responsiveness to treatment in RA in studies with tocilizumab—another IL-6R inhibitor—was proven to be predicted by certain single nucleotide polymorphisms (SNPs) in the IL6R gene [32,33,34]. While there are no studies about the influence of SNPs in the IL6R gene on the response to sarilumab in RA patients, it can be assumed that IL6R SNPs also play a role in the efficacy of sarilumab and might explain the occurrence of nonresponders. Of particular interest, especially concerning our in vitro results, is the relatively common SNP (approx. 35% in Caucasians) rs2228145 (formerly rs8192284) [31]. This SNP confers increased proteolytic conversion rates by the A Disintegrin and metalloproteinase domain (ADAM) proteases which in turn lead to higher serum levels of sIL-6R [35]. However, in the study by Enevold et al. (2014), rs2228145 alone had less impact on the efficacy of tocilizumab treatment [34]. A nonresponsive donor group can also be assumed in our in vitro study. These osteoblastic donor cells were not affected by IL-6/sIL-6R stimulation or the additional treatment with sarilumab. At the same time, it can be noted that the sarilumab-responsive donors showed an IL-6-dependent induction of ALP activity. The inconsistency of IL-6 responsiveness in studies with osteoblasts in vitro was already reported previously [3]. Apart from the donor-dependent effects in human primary cells, the use of osteoblasts of murine-origin or osteosarcoma-derived cells in the different in vitro studies may also account for the controversial results [3,27,36]. As the ability of a given cell type to respond to IL-6 strictly depends on the presence of the IL-6R α subunit, the absence of this subunit in some of the tested cell lines might explain the differing results [24].

Bone development and osteogenesis are significantly regulated by the Wnt-signaling pathway. This signaling pathway controls all steps of osteoblast differentiation. Excessive bone growth is antagonized by various physiological factors that interfere with the canonical Wnt receptor Frizzle and with LRP5 or LRP6 [37]. Bone loss in RA patients results from increased osteoclast-mediated resorption and decreased new bone formation. In the highly inflammatory context, bone resorption is not compensated by bone formation. Furthermore, the blockage of pro-osteoclast cytokines such as RANKL, TNF-α, and IL-6 did not reverse the inhibition of new bone formation in RA patients [37]. A study by Terpos et al. (2011) reported that female RA patients with high or moderate disease activity had significantly elevated serum levels of Dkk-1, sclerostin, and C-terminal cross-linking telopeptide of collagen type-I (CTX), while the osteoprotegerin/RANKL ratio was decreased compared to age-matched healthy, nonosteopenic women [38]. The alterations in the bone-remodeling markers are indicative of bone erosion in RA, and treatment with tocilizumab was able to attenuate these detrimental changes. The only exception was the level of sclerostin in RA patients. Treatment with tocilizumab in the patients even further elevated serum levels of sclerostin, a Wnt inhibitor. The authors speculated that there could be “a specific, beyond inflammation, effect of anti-IL-6 therapy on bone remodeling in humans” [38]. Although the influence of IL-6 on Wnt signaling is not clear and seems to depend on the sIL-6R level [39], the results of our study support a regulatory effect in Wnt signaling by IL-6 antagonism with sarilumab. As an example, transcriptome analysis of IL-6-responsive osteoblast donors showed the induction of DKK1 (2.14-fold) and DKK4 (2.01-fold) with concomitant inhibition of SFRP1 and SFRP4. In addition, receptor blockade by sarilumab had a regulatory effect on specific matrix proteins and thus on bone matrix maintenance. Consequently, sarilumab already influences downstream processes of new bone formation in the physiological milieu. Simultaneously, gene expression of MMP13, a protease that is secreted by osteoblasts and causes the degradation of collagen 1 [40], was reduced after supplementation with sarilumab. Because MMP13 also promotes cartilage matrix degradation in RA [41], sarilumab not only has a regulatory effect on bone resorption processes but may also have a regulatory function in the inflammatory bone-cartilage interaction of RA patients. Despite the suppression of the gene expression of proteins that play a role in regulating bone mass, there were no differences in mineralization after 14 days of stimulation or inhibition.

Despite the osteotropic effects of IL-6 on osteoblasts in bone formation, IL-6 also promotes bone resorption by activating osteoclasts. A study by Di Pompo et al. (2021) suggests that this is an osteoblast-mediated effect which is initiated by soluble mediators released from IL-6-activated osteoblasts [42]. In their study, the induction of osteoclast activity by osteoblast-conditioned medium was only suppressed when tocilizumab was added to the osteoblast culture, but not when it was added directly to the osteoclasts. It is most likely that RANKL is responsible for the observed effect [43]. Indeed, McGregor et al. (2019) state that osteoclast differentiation is not induced by direct action of IL-6 on osteoclast progenitors but is mediated via IL-6 trans-signaling in osteoblasts. The assumption that IL-6R inhibitors such as tocilizumab and sarilumab suppress bone resorption by decreasing RANKL expression in osteoblasts correlates well with the data from studies with RA patients. Indeed, our transcriptome analysis also showed that the transcripts of TNFS11 (RANKL) were downregulated by a factor of 1.47 in the sarilumab-responsive group. However, because the changes were minor (below the cut-off of a 2-fold change), we did not determine sRANKL in the cell culture supernatants. Clinical studies in RA patients, that determined serum markers of bone remodeling, reported beneficial effects with significantly reduced serum levels of sRANKL, an improved sRANKL/OPG ratio, and a trend of increased serum level of osteocalcin after treatment with sarilumab [16]. Treatment with tocilizumab not only decreased markers of bone resorption (CTX and C-terminal crosslinking telopeptide of type I collagen generated by MMPs (ICTP)) in RA patients, but also significantly increased serum levels of markers for bone formation such as osteocalcin and procollagen type I N-propeptide (PINP) [44].

In these studies, sarilumab or tocilizumab was tested in combination with methotrexate compared to a control group that received methotrexate plus placebo. It must be noted that methotrexate itself has a detrimental effect on bone mineral density [45,46]. Furthermore, contrary to the study of Terpos et al. (2011) [38], specific regulators of Wnt signaling, particularly sclerostin, were not determined [16,44]. This makes it difficult to assess whether the in vivo effect of IL-6R blockers on bone metabolism is solely beneficial. Sclerostin did not only react differently to other bone-remodeling markers in the study by Terpos et al. (2011) after IL-6 blockade, it is also supposed to be the key protein in the indirect regulation of bone formation by IL-6 via osteocytes [25]. Thus, the overall effect of the administration of sarilumab on bone metabolism in RA patients remains to be further investigated.

## 5. Conclusions

In conclusion, it was shown that, while effects of IL-6/IL-6R supplementation were mild, the highest tested concentrations of IL-6 and sIL-6R can significantly induce pro-osteogenic markers such as ALP activity after a stimulation period of 72 h. IL-6 receptor blockade for 72 h led to a reduction in regulatory proteins of important intracellular signaling pathways as well as mediators required for bone matrix maintenance including MGP and SPP1 which encode matrix Gla protein and osteopontin, two proteins that are of particular importance in the regulation of skeletal mineralization [47]. While reductions in those proteins might indicate that the administration of sarilumab exerts later effects on bone mineralization, there were no differences among control, stimulation, and inhibition of IL-6 signaling concerning the mineralization capacity after longer observation intervals of up to 14 days. Thus, the long-term mineralization experiments suggest that sarilumab might not exert a detrimental effect on bone formation.

The extent to which these effects may significantly influence bone formation or bone resorption in the highly inflammatory context of RA patients cannot be derived from our study, as we used only one proinflammatory cytokine in our rather static environment with IL-6 and sIL-6R. Therefore, further studies, e.g., in a highly inflammatory milieu with additional proinflammatory mediators, are required to investigate the interplay between IL-6R inhibition by sarilumab and the extent of its effects on crucial intracellular signaling pathways involved in bone homeostasis. For this purpose, the investigation of the effects in specific co-cultures with relevant cells may generate meaningful results, as intercellular communication is a major factor in the induction of intracellular signaling pathways. Further investigations with osteoblasts from RA patients must be performed to specifically demonstrate the clinical significance of IL-6R inhibition on bone-remodeling processes in the context of RA patients.

## Figures and Tables

**Table 1:** cDNA target sequences for semiquantitative real-time PCR. Primer pairs were purchased from Merck KGaA (Darmstadt, Germany).

Gene	Forward Primer (5′–3′)	Reverse Primer (5′–3′)
Alkaline phosphatase (ALP)	CATTGTGACCACCACGAGAG	CCATGATCACGTCAATGTCC
Integrin-binding sialoprotein (IBSP)	ATTTTGGGAATGGCCTGTGC	GTCACTACTGCCCTGAACTGG
Collagen type 1 (COL1A1)	ACGAAGACATCCCACCAATC	AGATCACGTCATCGCACAAC
Glycoprotein 130 (IL6ST)	ACTGTACAACTCGTGTGGAAGAC	TGCTCTCTGCTAAGTTCCCTTG
Hypoxanthine-guanine phosphoribosyltransferase (HPRT)	CCCTGGCGTCGTGATTAGTG	TCGAGCAAGACGTTCAGTCC
Interleukin 1β (IL1β)	TACTCACTTAAAGCCCGCCT	ATGTGGGAGCGAATGACAGA
Interleukin 6 (IL6)	TGGATTCAATGAGGAGACTTGCC	CTGGCATTTGTGGTTGGGTC
Interleukin 8 (IL8)	TCTGTGTGAAGGTGCAGTTTTG	ATTTCTGTGTTGGCGCAGTG
Matrix metalloprotease 13 (MMP13)	CACGCATAGTCATATAGATACT	CTGGAGATATGATGATACTAAC
Interleukin 6 receptor (IL6R)	CTCCTCTGCATTGCCATTGT	TGTGGCTCGAGGTATTGTCA
Osteopontin (SPP1)	AACGCCGACCAAGGAAAACT	GCACAGGTGATGCCTAGGAG

**Table 2:** Concentrations of soluble IL-6 receptor (sIL-6R) and soluble gp130 (sgp130) secreted by human osteoblasts (passage 3) after a 72 h stimulation with different concentrations of IL-6 (*n* = 5 independent donors (female: *n* = 4, male: *n* = 1); mean age: 77.8 ± 4.4 year). Protein contents were analyzed via ELISA and related to total protein in the cell culture supernatants. All results are shown as median and minimum/maximum values.

IL-6	0 ng/mL	1 ng/mL	10 ng/mL	50 ng/mL
**sIL-6R** [ng/g]	1.0 (0.7; 2.3)	0.9 (0.4; 2.2)	0.9 (0.5; 1.6)	0.8 (0.3; 1.8)
**sgp130** [ng/g]	76 (39; 90)	43 (31; 122)	46 (40; 84)	41 (28; 94)

**Table 3:** Concentrations of the c-terminal propeptide of collagen 1 (C1CP; *n* = 12 independent donors in passage 3 (female: *n* = 8, male: *n* = 4); mean age: 67.3 ± 16.1 year) and osteopontin (*n* = 4 independent donor (female: *n* = 3, male: *n* = 1; mean age: 77.5 ± 5.1 year)—*n* = 5 independent donors (female: *n* = 4, male: *n* = 1; mean age: 77.8 ± 4.4 year) after stimulation with IL-6, sIL-6R, and sarilumab. Protein contents were analyzed via ELISA and related to total protein. All results are shown as median and minimum/maximum values.

	50 ng/mL sIL-6R			50 ng/mL sIL-6R + Sarilumab		
IL-6	0 ng/mL	10 ng/mL	50 ng/mL	0 ng/mL	10 ng/mL	50 ng/mL
**C1CP** [ng/mg]	56 (29; 85)	56 (30; 75)	55 (36; 84)	55 (35; 111)	48 (32; 122)	54 (35; 94)
**OPN** [ng/g]	31 (18; 64)	19 (18; 52)	29 (0; 59)	0 (0; 192)	21 (0; 58)	17 (0; 302)

## Data Availability

All generated data are available within this manuscript.

## Supplementary Materials

The following supporting information can be downloaded at: https://www.mdpi.com/article/10.3390/pharmaceutics14071390/s1. Figure S1. Differentiation capacity of human primary osteoblasts in passage 4. (a) Alkaline phosphatase stain of monolayer cells. Red cells are positive for alkaline phosphatase (bar: 200 µm). (b) Gene expression profile of *Col1A1*, *ALP* and *BGLAP* in unstimulated osteoblasts. Results were calculated by 2^−ΔCt^ method (% housekeeping gene HPRT). Data are presented as box plots (*n* = 6). Figure S2. Course of gene (a–e) and protein expression (f) during in vitro cultivation of primary human osteoblasts from isolation up to passage (P) 3. Expression of genes related to the differentiation capacity of human primary osteoblasts—*Col1A1* (a), *ALP* (b), and *BGLAP* (c)—as well as of genes related to IL-6 signaling—*IL6ST* (d) and *IL6* (e)—were analyzed in osteoblasts directly after isolation and in passages 1, 2, and 3. Results were calculated by 2^−ΔCt^ method (% housekeeping gene HPRT). Release of IL-6 protein (f) into cell culture supernatant was determined in passages 1, 2, and 3. IL-6 protein was normalized to total protein release. Data are presented as box plots (*n* = 3–6). Statistical significance was determined via ANOVA with Bonferroni’s post hoc test: ** *p* < 0.01; *** *p* < 0.001. Figure S3. Correlation analysis of donor age to alkaline phosphatase (ALP) activity normalized to metabolic activity after exposure to IL-6 and sIL-6R (a) and to the difference (Δ) in ALP activity related to metabolic activity after incubation with and without sarilumab (b) for 72 h (*n* = 12 independent donors in passage 3 (female: *n* = 8, male: *n* = 4). All data are shown as single data points with linear regression and confidence intervals (95%). Female donors (green dot and purple square) and male donors (green up-facing triangle and purple down-facing triangle) were assigned separate symbols. Statistical analysis was performed with Pearson’s correlation analysis. The correlation coefficient r and the *p*-value of the analyses are reported in the graphs. Figure S4. Comparison of female and male donors for osteoblastic viability and alkaline phosphatase (ALP) activity after exposure to IL-6, sIL-6R ± sarilumab for 72 h (*n* = 8 independent female donors and *n* = 4 independent male donors, both in passage 3). (a) Alkaline phosphatase (ALP) activity of osteoblasts, which was quantified via hydrolysis of *p*-nitrophenyl phosphate. (b) Cell viability was tested via water-soluble tetrazolium salt (WST-)1 assay. (c) ALP activity related to metabolic activity. All data are shown as single data points with mean ± SD. Statistical significance was determined via repeated-measures two-way ANOVA with Bonferroni’s post hoc test. There were no significant differences. Figure S5. Comparison of female and male donors for mineralization capacity of osteoblasts after exposure to IL-6, sIL-6R ± sarilumab (*n* = 3 independent female donors [dots] and *n* = 3 independent male donors [triangles]). Cells were stimulated with IL-6, sIL-6R ± sarilumab over a period of 7 or 14 days in calcium-supplemented medium. Afterward, deposition of the hydroxyapatite portion of bone-like nodules was quantified via OsteoImage™. Fluorescence signals of mineralization were detected at excitation/emission wavelengths of 492/520 nm using a microplate reader. All data are shown as single data points with mean ± SD. Statistical significance was determined via repeated-measures two-way ANOVA with Bonferroni’s post hoc test.
