# Supplementary material for: Effects of the Interleukin-6 Receptor Blocker Sarilumab on Metabolic Activity and Differentiation Capacity of Primary Human Osteoblasts
Source: Pharmaceutics. 2022 Jun 30;14(7):1390. doi: 10.3390/pharmaceutics14071390 (PMC9318132; doi:10.3390/pharmaceutics14071390)
Supplement: Supplementary file 1 [file pharmaceutics-14-01390-s001.zip › pharmaceutics-1716600-supplementary.pdf]

Supplementary Materials: Effects of the Interleukin-6 Receptor Blocker Sarilumab on Metabolic Activity and Differentiation Capacity of Primary Human Osteoblasts

Annett Klinder, Janine Waletzko-Hellwig, Marie-Luise Sellin, Anika Seyfarth-Sehlke, Markus Wolfien, Franziska Prehn, Rainer Bader and Anika Jonitz-Heincke

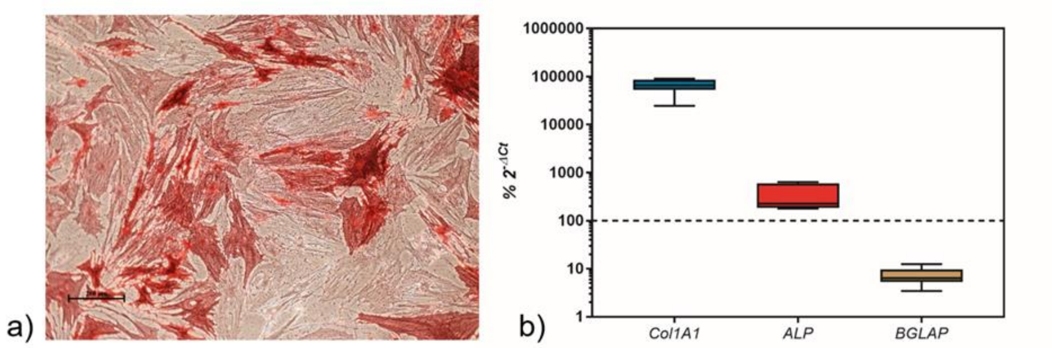

Figure S1. Differentiation capacity of human primary osteoblasts in passage 3. (**a**) Alkaline phosphatase stain of monolayer cells. Red cells are positive for alkaline phosphatase (bar: 200 µm). (**b**) Gene expression profile of Col1A1, ALP, and BGLAP in unstimulated osteoblasts. Results were calculated by $2^{-\Delta Ct}$ method (% housekeeping gene HPRT). Data are presented as boxplots (n = 6).

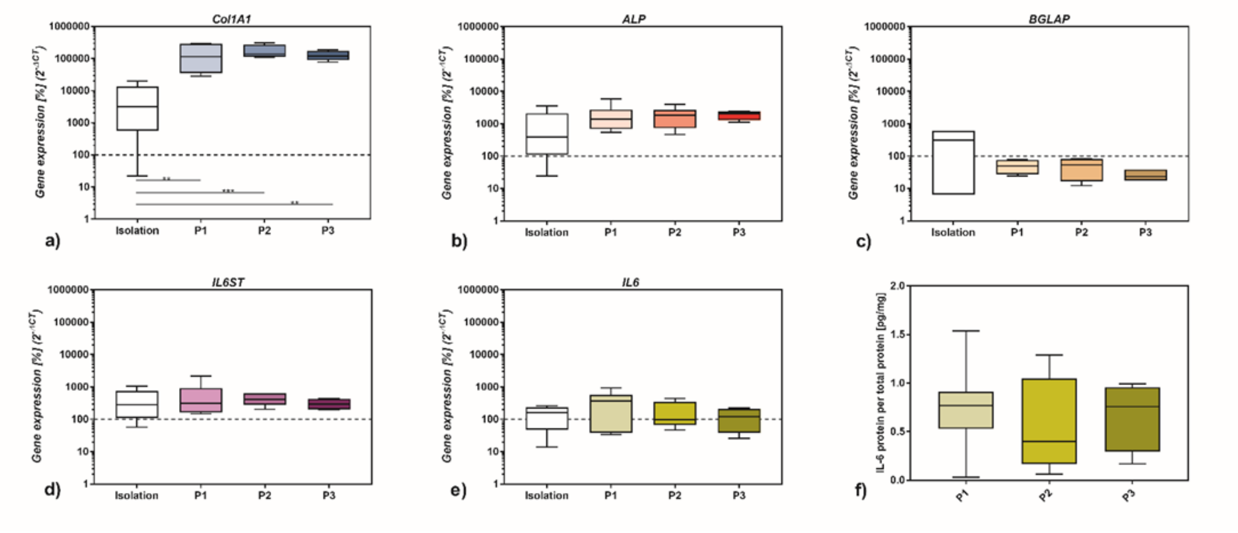

Figure S2. Course of gene (**a**–**e**) and protein expression (**f**) during in vitro cultivation of primary human osteoblasts from isolation up to passage (P) 3. Expression of genes related to the differentiation capacity of human primary osteoblasts—Col1A1 (**a**), ALP (**b**), and BGLAP (**c**)—as well as of genes related to IL-6 signaling—IL6ST (**d**) and IL6 (**e**)—were analyzed in osteoblasts directly after isolation and in passages 1, 2, and 3. Results were calculated by $2^{-\Delta Ct}$ method (% housekeeping gene HPRT). Release of IL-6 protein (**f**) into cell culture supernatant was determined in passages 1, 2, and 3. IL-6 protein was normalized to total protein release. Data are presented as box plots (n = 3–6). Statistical significance was determined via ANOVA with Bonferroni's post hoc test: ** $p < 0.01$; *** $p < 0.001$.

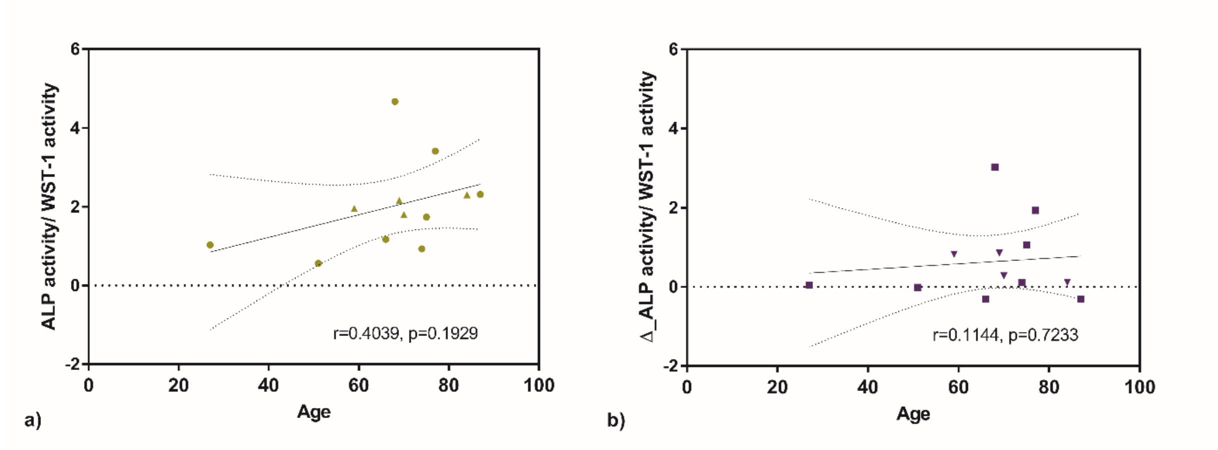

Figure S3. Correlation analysis of donor age to alkaline phosphatase (ALP) activity normalized to metabolic activity after exposure to IL-6 and sIL-6R (**a**) and to the difference (Δ) in ALP activity related to metabolic activity after incubation with and without sarilumab (**b**) for 72 h (n = 12 independent donors in passage 3 (female: n = 8, male: n = 4). All data are shown as single data points with linear regression and confidence intervals (95%). Female donors (green dot and purple square) and male donors (green up-facing triangle and purple down-facing triangle) were assigned separate symbols. Statistical analysis was performed with Pearson's correlation analysis. The correlation coefficient r and the p-value of the analyses are reported in the graphs.

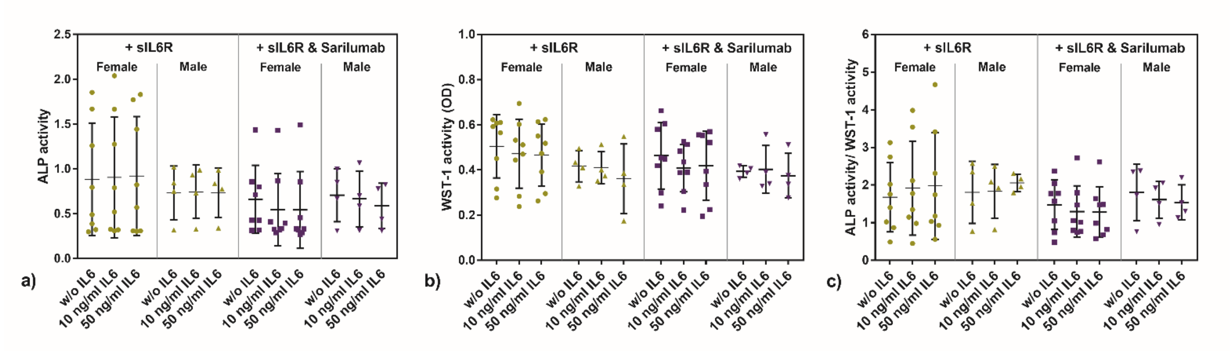

Figure S4. Comparison of female and male donors for osteoblastic viability and alkaline phosphatase (ALP) activity after exposure to IL-6, sIL-6R ± sarilumab for 72 h (n = 8 independent female donors and n = 4 independent male donors, both in passage 3). (**a**) Alkaline phosphatase (ALP) activity of osteoblasts, which was quantified via hydrolysis of p-nitrophenyl phosphate. (**b**) Cell viability was tested via water-soluble tetrazolium salt (WST-)1 assay. (**c**) ALP activity related to metabolic activity. All data are shown as single data points with mean ± SD. Statistical significance was determined via repeated-measures two-way ANOVA with Bonferroni's post hoc test. There were no significant differences.

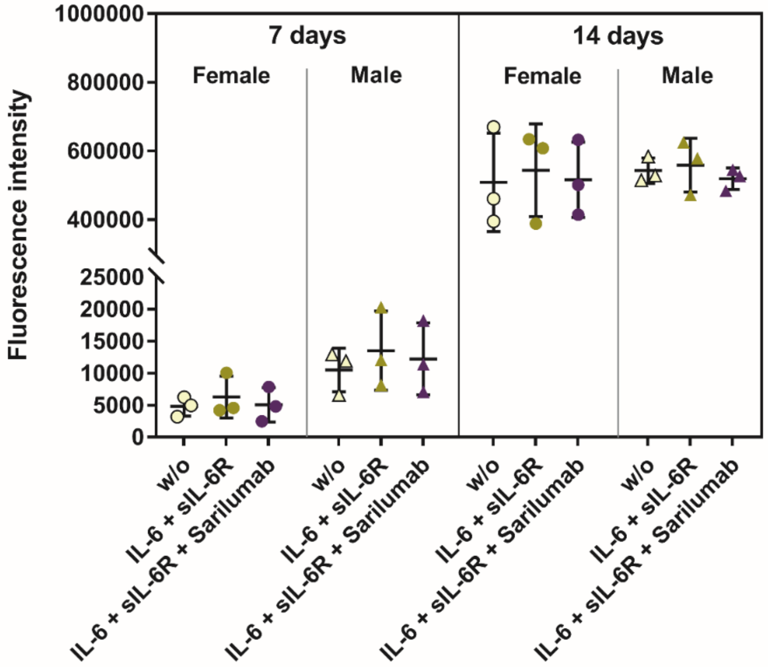

Figure S5. Comparison of female and male donors for mineralization capacity of osteoblasts after exposure to IL-6, sIL-6R ± sarilumab (*n* = 3 independent female donors [dots] and *n* = 3 independent male donors [triangles]). Cells were stimulated with IL-6, sIL-6R ± sarilumab over a period of 7 or 14 days in calcium-supplemented medium. Afterward, deposition of the hydroxyapatite portion of bone-like nodules was quantified via OsteoImage™. Fluorescence signals of mineralization were detected at excitation/emission wavelengths of 492/520 nm using a microplate reader. All data are shown as single data points with mean ± SD. Statistical significance was determined via repeated-measures two-way ANOVA with Bonferroni's post hoc test.
